# Detection and Characterization of *Paslahepevirus balayani* (Hepatitis E Virus) in Dairy Products from Hebei Province, China

**DOI:** 10.3390/pathogens14060564

**Published:** 2025-06-05

**Authors:** Xinyue Hu, Jinfeng Wang, Yinuo Wang, Wanzhe Yuan, Jianchang Wang, Xiangdong Xu

**Affiliations:** 1 School of Public Health, Hebei Medical University, Shijiazhuang 050017, China; hxy701012@126.com (X.H.); wangyinuo0618@126.com (Y.W.); 2 Food Microbiology and Animal Quarantine Laboratory, Technology Center of Shijiazhuang Customs, Shijiazhuang 050051, China; jinfengwang0625@163.com; 3 Hebei Key Laboratory of Environment and Human Health, Shijiazhuang 050017, China; 4 College of Veterinary Medicine, Hebei Agricultural University, Baoding 071001, China; yuanwanzhe@126.com

**Keywords:** *Paslahepevirus balayani*, zoonotic pathogen, sheep milk, goat milk, cross-species transmission

## Abstract

*Paslahepevirus balayani* (hepatitis E virus), a zoonotic pathogen transmitted primarily via the fecal–oral route, has undergone shifting transmission dynamics in China, with foodborne and zoonotic routes becoming increasingly significant. To assess the potential risk of HEV transmission through dairy products, this study investigated HEV RNA presence in raw milk from cows, sheep, and goats in Hebei Province, China. From March 2024 to April 2025, we collected 102 cow milk, 18 sheep milk, and 59 goat milk samples, analyzing them using RT-qPCR, with positive samples confirmed by RT-Nested PCR and partial ORF2 sequencing. While no HEV RNA was detected in cow milk, 3/18 (16.67%) sheep milk and 1/59 (1.69%) goat milk samples tested positive. Phylogenetic analysis of two sheep-derived and one goat-derived HEV isolate showed 99.43–100% nucleotide identity to local swine HEV strains (HEV/HB-SJZ158/CHN/2021 and HEV/HB-CD28/CHN/2021), all clustering within genotype 4d, the dominant subtype in the region. This study provided the first evidence of HEV RNA in sheep and goat milk in Hebei Province, suggesting possible cross-species transmission from pigs to ruminants. These findings highlighted the need for further research on HEV transmission risks through dairy products and emphasize the importance of monitoring zoonotic HEV strains in food safety assessments.

## 1. Introduction

*Paslahepevirus balayani* (hepatitis E virus) is a zoonotic pathogen that causes hepatitis E and poses a significant threat to public health worldwide. The World Health Organization (WHO) estimated that 20 million people were infected with HEV globally, with approximately 3.3 million symptomatic cases and more than 40,000 deaths due to hepatitis E, and that one-third of the population was exposed to the potential risk of HEV infection [1]. HEV is endemic globally, with large-scale outbreaks in developing countries through water and fecal–oral routes, and mainly sporadic and clustered cases in industrialized countries [2]. From 2009 to 2019, China cumulatively reported 296,183 cases of hepatitis E, accounting for 2.12% of viral hepatitis in the country, and the incidence rate rose from 1.53/10^5^ in 2009 to 2.02/10^5^ in 2019 [3]. The Chinese statutory infectious disease report showed that the number of reported cases of hepatitis E exceeded hepatitis A in the last decade, and its mortality rate had risen to the first place among the five major viral hepatitides [4]. HEV infection is typically regarded as a self-limiting disease; however, acute hepatitis E could result in severe liver failure. Additionally, HEV infection may lead to chronic hepatitis and even cirrhosis, particularly in immunocompromised hosts who may endure more severe infections with a worse prognosis [5,6].

HEV is a positive-sense single-stranded RNA virus with a diameter of 27 to 34 nm, a genome length of about 7.2 kb [7,8], and it contains three or four open reading frames (ORFs) [9]. ORF1 encodes a non-structural protein; ORF2 encodes a viral capsid protein that is crucial for viral assembly and propagation; ORF3 encodes a functional ion channel; and a fourth open reading frame, ORF4, has been identified, which encodes a protein believed to enhance RdRp (RNA-dependent RNA polymerase) activity [9]. HEV is categorized into eight genotypes (HEV-1–8). HEV-1 and HEV-2 infect only humans, while HEV-3, HEV-4, and HEV-7 are zoonotic. In contrast, HEV-5, HEV-6, and HEV-8 infect only animals [10]. HEV-1–4 are more closely related to human infections [10]. HEV-1 and HEV-2 are primarily transmitted through the fecal–oral route and can also be spread through blood transfusions [11]. They often lead to large-scale outbreaks in low- and middle-income countries [12,13]. HEV-3 and HEV-4 are primarily found in industrialized countries, with HEV-3 being distributed worldwide and HEV-4 predominantly in Asia and Europe [14]. Since the first detection of HEV-4 in China in 1993, it had been reported in several different regions including Hebei, Shandong, Guangdong, and Shanghai [15,16], and it has been the dominant genotype in China recently [17]. HEV-4 infection is mainly transmitted by consuming contaminated food, particularly raw or undercooked meat. The primary host is pigs, but HEV RNA has also been detected in wild boars, cows, goats, sheep, deer, horses, raccoons, rabbits, cats, and dogs [18,19,20,21,22,23]. Consequently, these animals could be regarded as the potential zoonotic sources of transmission to humans.

The quality issues of milk and dairy products have long been widely concerned in the dairy industry and public health sector. However, there was increasing evidence that HEV was present in the milk of ruminants [24]. The research indicated that HEV in milk was infectious and could transmit to rhesus monkeys [23]. These findings raised considerable concerns regarding the risk of HEV transmission from milk and other ruminant dairy products to consumers. In recent years, the presence of HEV RNA in cow milk, sheep milk, and goat milk had been reported in countries such as the Czech Republic [24], Turkey [25], and Egypt [26]. Additionally, the presence of HEV RNA in cow milk and goat milk in rural areas of Yunnan Province, China, was also reported [27]. Considering the importance of milk and dairy products in human daily life, there is an urgent need for more in-depth research on the presence of HEV in milk.

In this study, HEV RNA was detected in cow milk, sheep milk, and goat milk from different regions of Hebei Province using RT-qPCR and RT-Nested PCR, and the phylogenetic tree of its gene sequence was constructed and analyzed.

## 2. Materials and Methods

### 2.1. Sample Collection

**Figure 1:**
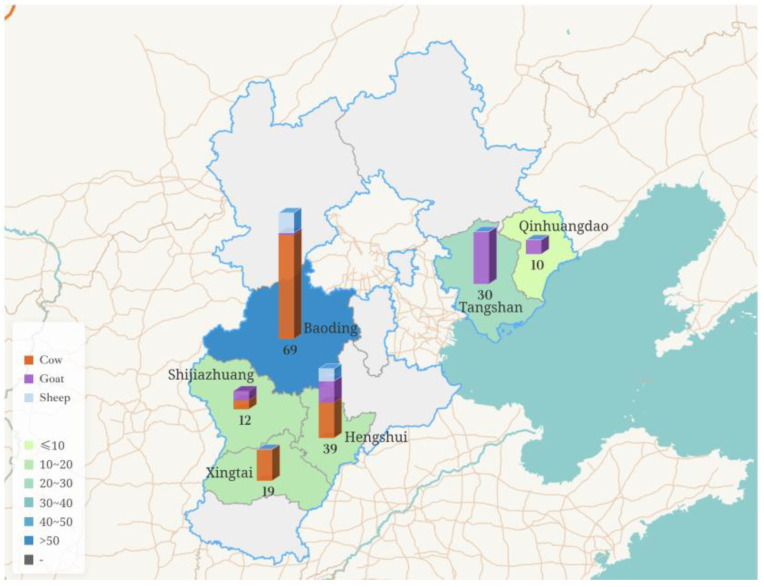
Profile map of Hebei Province, China, showing the variety of sample types and sampling volumes from different regions.

From March 2024 to April 2025, a total of 179 milk samples, including cow milk, sheep milk, and goat milk, were collected from Shijiazhuang, Baoding, Hengshui, Xingtai, Qinhuangdao, and Tangshan in Hebei Province, China (Figure 1). All samples were transported in insulated boxes with ice packs (4 ± 0.5 °C) to the laboratory for immediate processing or were stored at −80 °C until they were tested.

The partial sheep and goat milk samples were collected from farmers in rural areas who raised sheep or goats on a free-range basis, with each household typically keeping 1–2 lactating sheep or goats. These farmers regularly grazed their animals in forested areas and on farmland where no crops were grown. Six cow milk samples were collected from local street vendors. The other part of the milk samples are collected from large-scale farms that are strictly managed and undergo regular disinfection to ensure a relatively hygienic environment. The types of samples, their sources, and sample sizes are detailed in Table 1.

### 2.2. Viral RNA Extraction

One milliliter of each sample was collected and centrifuged at 6000× *g* for 5 min at 4 °C to isolate the liquid phase between the precipitate and the upper lipid layer. One hundred and forty microliters of the processed liquid phase were then used for the viral RNA extraction with a commercial viral RNA extraction kit (TianGen Biotech Co., Ltd., Beijing, China) according to the manufacturer’s instructions.

### 2.3. RT-qPCR and RT-Nested PCR

One-step RT-qPCR [28] was used to detect HEV RNA in the milk. The 25 μL reaction system consisted of 5 μL of 5× One Step RT-qPCR Master Mix (Sanshi Biotech, Shijiazhuang, China), 1 μL of Enzyme Mix, 1 μL of each primer (10 μmol/L), 0.5 μL of probe (10 μmol/L), 11.5 μL of RNase-Free ddH2O, and 5 μL of extracted RNA. RT-qPCR was performed on a Quant Studio 5 (Thermo Fisher Scientific, Waltham, CA, USA) under the following reaction conditions: reverse transcription at 50 °C for 15 min, followed by denaturation at 95 °C for 5 s, and immediate amplification of 40 cycles at 95 °C for 5 s and at 60 °C for 30 s. The RT-qPCR-positive RNA was reverse-transcribed using the Prime Script II 1st Strand cDNA Synthesis Kit (Thermo Fisher Scientific, Waltham, CA, USA). The gene fragment of the ORF2 region was amplified by an RT-Nested PCR [29]. The above primers and probe information are provided in Table 2, and they were synthesized by Shanghai Generay Biotech Co., Ltd. (Shanghai, China).

### 2.4. Sequencing and Phylogenetic Analysis of the HEV ORF2 Gene

The RT-Nested PCR products were purified using an agarose gel DNA recovery kit (Sanshi Biotechnology, Shijiazhuang, China), cloned into the pTOPO-TA vector (Beijing Aidlab Biotechnology, Beijing, China) and transformed into DH5α for Sanger sequencing by a commercial company (Zhongke Xilin, Beijing, China).

The obtained nucleotide sequences were aligned for homology analysis with other known HEV sequences in GenBank on the NCBI (National Center for Biotechnology Information) (https://www.ncbi.nlm.nih.gov (accessed on 11 February 2025)). Using the ML (Maximum Likelihood) method in MEGA 11.0 software, a phylogenetic tree was constructed with the obtained nucleotide sequences and 42 reference sequences of different HEV genotypes. The phylogenetic tree was labeled and refined using the online tool tvBOT [30]. The HEV reference sequences were sourced from the GenBank database. The standard classification of HEV genotypes and subtypes was based on the research of Smith and others [10].

## 3. Results

### 3.1. HEV RNA in Cow, Sheep, and Goat Milk

A total of 179 milk samples were analyzed for the presence of HEV RNA using an RT-qPCR assay, and 4 samples were positive for HEV RNA, comprising 3 sheep milk samples and 1 goat milk sample. Overall, the positive rate for sheep and goat milk was 5.19% (4/77). Specifically, three sheep milk samples (16.67%, 3/18) were positive, with two positive samples collected in Baoding and one in Hengshui. Only one goat milk sample (1.69%, 1/59) was positive, which was also from Hengshui. Notably, HEV RNA was not detected in any cow milk samples. Detailed information regarding these findings is presented in Table 3.

### 3.2. Analysis of Sequencing Results

RT-Nested PCR was conducted on the four HEV RNA-positive milk samples to amplify the partial ORF2 fragment of HEV. The expected 348 bp RT-Nested PCR products were successfully obtained from two sheep milk samples (HEV-YN3 and HEV-YN8) and one goat milk sample (HEV-YN24).

Through a blast comparison with the HEV ORF2 fragment sequences available in GenBank, the results indicated that HEV-YN24 shared 100% identity with the swCH25 (AY594199), HEV/HB-SJZ158/CHN/2021 (ON152326), and HEV/HB-CD28/CHN/2021 (ON152328) isolates, while HEV-YN3 and HEV-YN8 showed 99.43% identity with the swCH25, HEV/HB-SJZ158/CHN/2021, and HEV/HB-CD28/CHN/2021 isolates. The isolate swCH25 originates from Xinjiang, China, while HEV/HB-SJZ158/CHN/2021 and HEV/HB-CD28/CHN/2021 are both from Hebei Province, China. Furthermore, the three HEV isolates from sheep and goat milk had 91.06% and 89.74% identity with human isolates BDM30 (KF691587) and BDJ2 (KF691590) from Baoding City in Hebei Province, respectively.

The sequences have been uploaded to the GenBank database with the accession numbers PQ394076 (HEV-YN3), PQ394077 (HEV-YN8), and PQ394078 (HEV-YN24).

### 3.3. Phylogenetic Analysis

**Figure 2:**
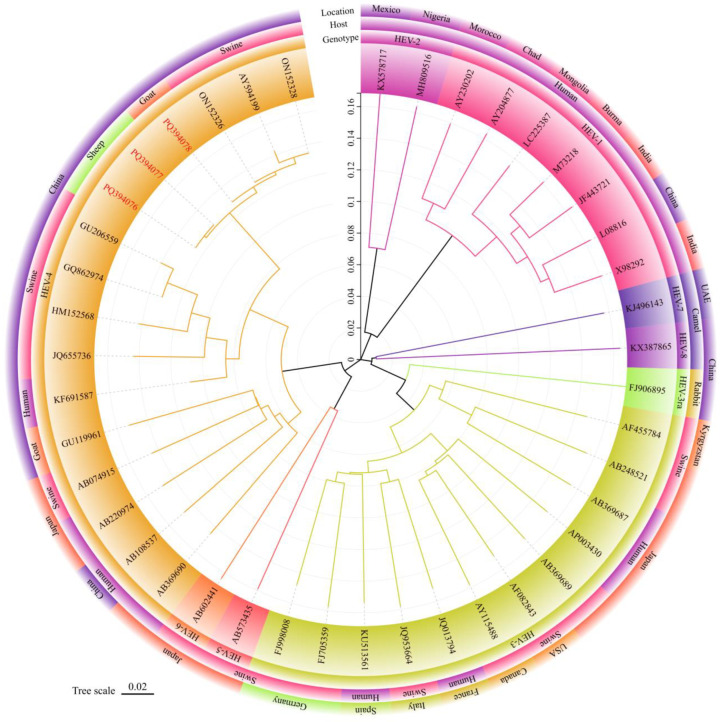
Phylogenetic tree for the 348 nt sequences within open reading frame 2 of hepatitis E viruses in milk from sheep and goats. The sequence of HEV strains in this study was indicated by red Accession. Different colored blocks represent the genotypes, hosts, and location of different strains. The evolutionary history was inferred using the Maximum Likelihood method and General Time Reversible model. The optimal tree was shown. Evolutionary analyses were conducted in MEGA 11.0 [31].

The phylogenetic tree was constructed using the ML method of MEGA 11.0 software (Figure 2), and the reliability of the phylogenetic tree was evaluated using bootstrap 1000 replicates. The phylogenetic analysis indicated that the three HEV isolates identified in this study all belonged to the HEV-4d subtype, clustering in the known HEV-4d branch alongside the pig-derived HEV isolates HEV/HB-SJZ158/CHN/2021, HEV/HB-CD28/CHN/2021 from Hebei Province, and the Xinjiang pig-derived HEV isolate swCH25.

## 4. Discussion

HEV is one of the most common pathogens of acute viral hepatitis worldwide, and the number of HEV infections has been increasing in recent years. Given the high prevalence of HEV infection and its progressively expanding host range, more scholars are focusing on the zoonotic nature of HEV and the close association between specific animal species and humans. Recent reports have documented multiple cases in which human HEV demonstrated a highly consistent genomic sequence with viruses isolated in local animals [32]. Previous studies have detected HEV RNA in animal liver and fecal samples [32,33]. Increasing evidence showed that HEV had been detected in the milk of various mammals, including humans, suggesting that HEV might be transmitted through milk [23,27,34].

In this study, HEV RNA was detected in three sheep milk and one goat milk samples, which was the first report of the presence of HEV in sheep and goat milk in Hebei Province, China. The results were consistent with the studies of Dziedzinska [24], Demirci [25] (some confirmation is still necessary), and El-Mokhtar [35], in which HEV RNA was also detected in sheep or goat milk. Notably, HEV RNA was detected in both sheep milk and goat milk, indicating that sheep milk and goat milk might increase the risk of HEV transmission to humans through the food chain. Phylogenetic analysis showed that all newly acquired HEV strains clustered within the known HEV-4d branch, belonging to the HEV-4d subtype, which was consistent with the fact that HEV-4d was the main genetic subtype of animal-derived and human-derived HEV in Hebei Province [16,36,37]. HEV-YN3, HEV-YN8, and HEV-YN24 were found to be on the same branch as the pig-derived HEV isolates HEV/HB-SJZ158/CHN/2021 and HEV/HB-CD28/CHN/2021 from Hebei Province and the pig-derived HEV isolate swCH25 from Xinjiang. The 99.43–100% nucleotide sequence identity between the swine-derived HEV strains and the HEV strains in this study indicated the potential cross-species transmission of HEV among pigs, sheep, and goats in Hebei Province. The HEV isolates obtained from goat feces and goat milk by Di Martino et al. [38] in Italy and by El-Mokhtar et al. [35] in Egypt were HEV-3. In contrast, the HEV isolates obtained from sheep and goat milk by Demirci et al. [25] in Turkey included HEV-1, HEV-3, and HEV-4. Additionally, the HEV isolates from goat milk by Long et al. [27] in China were HEV-4. The genotype of the HEV obtained from sheep and goat milk in this study was also HEV-4. The difference in HEV genotypes within the same host might be related to varying geographic environments [11]. HEV-4 is the main circulating genotype in China and several Southeast Asian countries including Indonesia, Cambodia, Thailand, Vietnam, Laos, and Myanmar [39], while Europe predominantly reported HEV-3 [40,41].

HEV RNA was not detected in any of the 102 cow milk samples from Hebei Province in this study, which was different from the results in the studies of Huang et al. [23] in China, Demirci et al. [25] in Turkey, and Sayed et al. [26] in Egypt. However, our results were consistent with the results of Baechlein et al. [42] in Germany, Vercouter et al. [43] in Flanders, and Geng et al. [44] in China, in which HEV RNA was also not detected in cow milk. The above differences might relate to factors such as geographic location, sanitation levels, and farming practices. Furthermore, the significant inconsistencies in the results obtained from different testing methods also hindered the interpretation of the actual differences in HEV infection rates across various studies.

Hebei Province, located in the north of China, is a large agricultural province with a tradition of raising meat animals such as pigs, sheep, and goats in rural areas. HEV is widely prevalent among animals like pigs and rabbits in the province [45]. It had been demonstrated that the large-scale farming model largely reduced the exposure risk of animals to environmental HEV, whereas the rural free-range farming model could greatly increase the exposure risk. The dairy cow farms and two goat farms in this study had relatively strict disinfection measures, which might have reduced the contact between the animals and viruses in the surrounding environment to some extent. Furthermore, the dairy cow farms and two goat farms in this study focused on single-species farming, thereby minimizing the risk of HEV cross-species transmission. Sheep and goats in rural regions are mainly grazed in woodlands or fields, and the manure produced, especially by pigs, is typically composted for use as fertilizer in agricultural fields, making it easy for sheep and goats to become infected with HEV through the fecal–oral route. When grazing outdoors, livestock come into contact with vectors such as rodents, wild birds, and insects, as well as with different types of animals, which increases the likelihood of HEV transmission both within and across species [23]. The above facts also supported the results in this study of all four positive milk samples collected from the rural free-range model.

The prevalence and molecular characteristics of HEV in cow, sheep, and goat milk in Hebei Province were preliminarily analyzed in this study, providing new epidemiological data on HEV infections. The results reflected the prevalence of HEV in rural sheep and goats in Hebei and further confirmed that sheep and goats might be important hosts for HEV. Notably, there was a high identity among the nucleotide sequences of HEV isolates from pigs, sheep, and goats in Hebei, indicating the potential cross-species transmission of HEV among livestock. Meanwhile, several drawbacks were presented in this study, such as the low number of samples collected and the different regions in Hebei Province. Future studies should focus on monitoring HEV in different milk and dairy products from a wide range of ruminants and assessing the risk of the zoonotic transmission of HEV from milk products to humans.

## 5. Conclusions

HEV was identified in sheep and goat milk in Hebei Province for the first time, with the genetic subtype classified as HEV-4d, which is the main circulating HEV subtype in northern China. The homology analysis indicated the possibility of the cross-species transmission of HEV among pigs, sheep, and goats. The results could enhance our understanding of HEV prevalence and aid in the development of strategies to mitigate the risk of HEV infection in humans.

## Figures and Tables

**Table 1:** The details of the ruminant milk samples used in this study.

Location	Animal Species	Breeds	Area	Number of Samples	Total
Shijiazhuang	Cow	Holstein Cow	Street vendors	6	12
	Goat	Boer Goat	Rural	6	
Baoding	Cow	Holstein Cow	Farms	57	69
	Goat	Boer Goat	Rural	1	
	Sheep	Small-Tailed Han Sheep	Rural	11	
Hengshui	Cow	Holstein Cow	Farm	20	39
	Goat	Boer Goat	Rural	12	
	Sheep	Small-Tailed Han Sheep	Rural	7	
Xingtai	Cow	Holstein Cow	Farm	19	19
Qinhuangdao	Goat	Cashmere Goat	Farm	10	10
Tangshan	Goat	Saanen Goat	Farm	30	30
Total	-	-	-	-	179

**Table 2:** Primers and probes for RT-qPCR and RT-Nested PCR used in this study ^1^.

Amplified Region	Primer/Probes Designation	Sequences (5′-3′)	Nucleotide Region Spanning	Product Length (bp)	Reference
ORF3	JVHEVF	GGTGGTTTCTGGGGTGAC	5310–5327	70	[28]
	JVHEVR	AGGGGTTGGTTGGATGAA	5362–5379		
	JVHEVP	FAM-TGATTCTCAGCCCTTCGC-MGB	5333–5350		
ORF2	3156NF	AATTATGCYCAGTAYCGRGTTG ^2^	5736–5757	731	[29]
	3157NR	CCCTTRTCYTGCTGMGCATTCTC ^2^	6444–6466		
	3158NF	GTWATGCTYTGCATWCATGGCT ^2^	6021–6040	348	
	3159NR	AGCCGACGAAATCAATTCTGTC	6347–6366		

^1^ The reference strain accession number for RT-qPCR is AY594199.1 and that for RT-Nested PCR is GU206559.1. ^2^ M = A or C; R = A or G; W = A/T; Y = C or T.

**Table 3:** HEV RNA detection results of the milk samples.

Location	Cow	Goat	Sheep	Total
Shijiazhuang	0.00 (0/6)	0.00 (0/6)	-	0.00 (0/12)
Baoding	0.00 (0/57)	0.00 (0/1)	27.27 (3/11)	4.35 (3/69)
Hengshui	0.00 (0/20)	8.33 (1/12)	0.00 (0/7)	2.56 (1/39)
Xingtai	0.00 (0/19)	-	-	0.00 (0/19)
Qinhuangdao	-	0.00 (0/10)	-	0.00 (0/10)
Tangshan	-	0.00 (0/30)	-	0.00 (0/30)
Total	0.00 (0/102)	1.69 (1/59)	16.67 (3/18)	2.23 (4/179)

Note: Positive rate % (number of positive samples/number of samples).

## Data Availability

The raw data supporting the conclusions of this article will be made available by the authors on request.

## References

[1] World Health Organization Hepatitis E. https://www.who.int/news-room/fact-sheets/detail/hepatitis-e.

[2] Yugo D.M., Meng X.J. (2013). Hepatitis E virus: Foodborne, waterborne and zoonotic transmission. Int. J. Environ. Res. Public Health.

[3] Dong D., Zou S., Tang S. (2024). Analysis on the spatial-temporal epidemiological characteristics of viral hepatitis in China from 2009 to 2019. Mod. Prev. Med..

[4] National Disease Control and Prevention Administration of China Overview of the Epidemic Situation of Statutory Infectious Diseases in the Country for 2024. https://www.ndcpa.gov.cn/.

[5] Park K., Kim J., Noh J., Kim K., Yang E., Kim S.G., Cho H.K., Byun K.S., Kim J.H., Lee Y.S. (2024). First detection and characterization of hepatitis E virus (*Rocahepevirus ratti*) from urban Norway rats (*Rattus norvegicus*) in the Republic of Korea. J. Med. Virol..

[6] Kamar N., Selves J., Mansuy J.M., Ouezzani L., Péron J.M., Guitard J., Cointault O., Esposito L., Abravanel F., Danjoux M. (2008). Hepatitis E virus and chronic hepatitis in organ-transplant recipients. N. Engl. J. Med..

[7] Reyes G.R., Purdy M.A., Kim J.P., Luk K.C., Young L.M., Fry K.E., Bradley D.W. (1990). Isolation of a cDNA from the virus responsible for enterically transmitted non-A, non-B hepatitis. Science.

[8] Tam A.W., Smith M.M., Guerra M.E., Huang C.C., Bradley D.W., Fry K.E., Reyes G.R. (1991). Hepatitis E virus (HEV): Molecular cloning and sequencing of the full-length viral genome. Virology.

[9] Cancela F., Noceti O., Arbiza J., Mirazo S. (2022). Structural aspects of hepatitis E virus. Arch. Virol..

[10] Smith D.B., Izopet J., Nicot F., Simmonds P., Jameel S., Meng X.J., Norder H., Okamoto H., van der Poel W.H.M., Reuter G. (2020). Update: Proposed reference sequences for subtypes of hepatitis E virus (species *Orthohepevirus A*). J. Gen. Virol..

[11] Li S., Liu M., Cong J., Zhou Y., Miao Z. (2017). Detection and Characterization of Hepatitis E Virus in Goats at Slaughterhouse in Tai’an Region, China. Biomed. Res. Int..

[12] Kamar N., Izopet J., Pavio N., Aggarwal R., Labrique A., Wedemeyer H., Dalton H.R. (2017). Hepatitis E virus infection. Nat. Rev. Dis. Primers.

[13] Wang B., Meng X.J. (2021). Hepatitis E virus: Host tropism and zoonotic infection. Curr. Opin. Microbiol..

[14] Turlewicz-Podbielska H., Augustyniak A., Wojciechowski J., Pomorska-Mól M. (2023). Hepatitis E Virus in Livestock—Update on Its Epidemiology and Risk of Infection to Humans. Animals.

[15] Wang X.J., Zhao Q., Jiang F.L., Liu B.Y., Zhao J.N., Dang L., Sun Y.N., Mu Y., Xiao S.Q., Wang C.B. (2014). Genetic characterization and serological prevalence of swine hepatitis E virus in Shandong province, China. Vet. Microbiol..

[16] Wang K., Liu L., Wang J., Sun X., Han Q., Zhou C., Xu X., Wang J. (2023). Quantification of hepatitis E virus in raw pork livers using droplet digital RT-PCR. Food Microbiol..

[17] Wang W., Wu W., Chen M., Teng Z. (2025). Prevalence of hepatitis E virus in domestic animals in the Chinese mainland: A systematic review and meta-analysis. BMC Vet. Res..

[18] Doceul V., Bagdassarian E., Demange A., Pavio N. (2016). Zoonotic Hepatitis E Virus: Classification, Animal Reservoirs and Transmission Routes. Viruses.

[19] Schlosser J., Eiden M., Vina-Rodriguez A., Fast C., Dremsek P., Lange E., Ulrich R.G., Groschup M.H. (2014). Natural and experimental hepatitis E virus genotype 3-infection in European wild boar is transmissible to domestic pigs. Vet. Res..

[20] Izopet J., Dubois M., Bertagnoli S., Lhomme S., Marchandeau S., Boucher S., Kamar N., Abravanel F., Guérin J.L. (2012). Hepatitis E virus strains in rabbits and evidence of a closely related strain in humans, France. Emerg. Infect. Dis..

[21] Yan B., Zhang L., Gong L., Lv J., Feng Y., Liu J., Song L., Xu Q., Jiang M., Xu A. (2016). Hepatitis E Virus in Yellow Cattle, Shandong, Eastern China. Emerg. Infect. Dis..

[22] Tian F., Li J., Liu Y., Liu W., Liu Y., Xu S., Tong Y., Feng F. (2024). First molecular evidence of hepatitis E virus in farmed raccoon dogs. Emerg. Microbes Infect..

[23] Huang F., Li Y., Yu W., Jing S., Wang J., Long F., He Z., Yang C., Bi Y., Cao W. (2016). Excretion of infectious hepatitis E virus into milk in cows imposes high risks of zoonosis. Hepatology.

[24] Dziedzinska R., Krzyzankova M., Bena M., Vasickova P. (2020). Evidence of Hepatitis E Virus in Goat and Sheep Milk. Viruses.

[25] Demirci M., Yiğin A., Ünlü Ö., Kılıç Altun S. (2019). Detection of HEV RNA amounts and genotypes in raw milks obtained from different animals. Mikrobiyol. Bül..

[26] Sayed I.M., Elkhawaga A.A., El-Mokhtar M.A. (2020). Circulation of hepatitis E virus (HEV) and/or HEV-like agent in non-mixed dairy farms could represent a potential source of infection for Egyptian people. Int. J. Food Microbiol..

[27] Long F., Yu W., Yang C., Wang J., Li Y., Li Y., Huang F. (2017). High prevalence of hepatitis E virus infection in goats. J. Med. Virol..

[28] Jothikumar N., Cromeans T.L., Robertson B.H., Meng X.J., Hill V.R. (2006). A broadly reactive one-step real-time RT-PCR assay for rapid and sensitive detection of hepatitis E virus. J. Virol. Methods.

[29] Huang F.F., Haqshenas G., Guenette D.K., Halbur P.G., Schommer S.K., Pierson F.W., Toth T.E., Meng X.J. (2002). Detection by reverse transcription-PCR and genetic characterization of field isolates of swine hepatitis E virus from pigs in different geographic regions of the United States. J. Clin. Microbiol..

[30] Xie J., Chen Y., Cai G., Cai R., Hu Z., Wang H. (2023). Tree Visualization by One Table (tvBOT): A web application for visualizing, modifying and annotating phylogenetic trees. Nucleic Acids Res..

[31] Tamura K., Stecher G., Kumar S. (2021). MEGA11: Molecular Evolutionary Genetics Analysis Version 11. Mol. Biol. Evol..

[32] Wu J., Si F., Jiang C., Li T., Jin M. (2015). Molecular detection of hepatitis E virus in sheep from southern Xinjiang, China. Virus Genes.

[33] Xu F., Pan Y., Baloch A.R., Tian L., Wang M., Na W., Ding L., Zeng Q. (2014). Hepatitis E virus genotype 4 in yak, northwestern China. Emerg. Infect. Dis..

[34] Rivero-Juarez A., Frias M., Rodriguez-Cano D., Cuenca-López F., Rivero A. (2016). Isolation of Hepatitis E Virus from Breast Milk During Acute Infection. Clin. Infect. Dis..

[35] El-Mokhtar M.A., Elkhawaga A.A., Sayed I.M. (2020). Assessment of hepatitis E virus (HEV) in the edible goat products pointed out a risk for human infection in Upper Egypt. Int. J. Food Microbiol..

[36] Geng Y., Zhao C., Guo T., Xu Y., Wang X., Huang W., Liu H., Wang Y. (2019). Detection of Hepatitis E Virus in Raw Pork and Pig Viscera as Food in Hebei Province of China. Foodborne Pathog. Dis..

[37] Geng Y., Zhao C., Fan J., Harrison T.J., Zhang H., Lian H., Geng K., Wang Y. (2013). Genotype analysis of hepatitis E virus from sporadic hepatitis E cases in northern China. Infect. Genet. Evol..

[38] Di Martino B., Di Profio F., Melegari I., Sarchese V., Robetto S., Marsilio F., Martella V. (2016). Detection of hepatitis E virus (HEV) in goats. Virus Res..

[39] Khuroo M.S., Khuroo M.S., Khuroo N.S. (2016). Transmission of Hepatitis E Virus in Developing Countries. Viruses.

[40] Priemer G., Cierniak F., Wolf C., Ulrich R.G., Groschup M.H., Eiden M. (2022). Co-Circulation of Different Hepatitis E Virus Genotype 3 Subtypes in Pigs and Wild Boar in North-East Germany, 2019. Pathogens.

[41] Izopet J., Tremeaux P., Marion O., Migueres M., Capelli N., Chapuy-Regaud S., Mansuy J.M., Abravanel F., Kamar N., Lhomme S. (2019). Hepatitis E virus infections in Europe. J. Clin. Virol..

[42] Baechlein C., Becher P. (2017). No evidence for zoonotic hepatitis E virus infection through dairy milk in Germany. Hepatology.

[43] Vercouter A.S., Sayed I.M., Lipkens Z., De Bleecker K., De Vliegher S., Colman R., Koppelman M., Supré K., Meuleman P. (2018). Absence of zoonotic hepatitis E virus infection in Flemish dairy cows. Int. J. Food Microbiol..

[44] Geng Y., Zhao C., Huang W., Wang X., Xu Y., Wu D., Du Y., Liu H., Wang Y. (2019). Hepatitis E virus was not detected in feces and milk of cows in Hebei province of China: No evidence for HEV prevalence in cows. Int. J. Food Microbiol..

[45] Liu H., Geng K., Wang C., Shi T., Zhang H., Zhao C., Geng Y. (2024). Epidemiological study of hepatitis E virus infection among students and workers in Hebei Province of China. Zoonoses Public Health.

